# Show us the data: global COVID-19 wastewater monitoring efforts, equity, and gaps

**DOI:** 10.1093/femsmc/xtad003

**Published:** 2023-01-12

**Authors:** Colleen C Naughton, Fernando A Roman, Ana Grace F Alvarado, Arianna Q Tariqi, Matthew A Deeming, Krystin F Kadonsky, Kyle Bibby, Aaron Bivins, Gertjan Medema, Warish Ahmed, Panagis Katsivelis, Vajra Allan, Ryan Sinclair, Joan B Rose

**Affiliations:** Department of Civil and Environmental Engineering, University of California, Merced, Merced, CA 95343, United States; Department of Civil and Environmental Engineering, University of California, Merced, Merced, CA 95343, United States; Department of Civil and Environmental Engineering, University of California, Merced, Merced, CA 95343, United States; Department of Civil and Environmental Engineering, University of California, Merced, Merced, CA 95343, United States; Department of Civil and Environmental Engineering, University of California, Merced, Merced, CA 95343, United States; Department of Civil and Environmental Engineering, University of California, Merced, Merced, CA 95343, United States; Department of Civil and Environmental Engineering and Earth Science, University of Notre Dame, 156 Fitzpatrick Hall, Notre Dame, IN 46556, United States; Department of Civil and Environmental Engineering and Earth Science, University of Notre Dame, 156 Fitzpatrick Hall, Notre Dame, IN 46556, United States; KWR Water Research Institute, Groningenhaven 7, 3433 PE Nieuwegein, The Netherlands; Civil Engineering and Geosciences, Delft University of Technology, Stevinweg 1, 2628 CN Delft, The Netherlands; Michigan State University, 1405 S Harrison Rd, East-Lansing, MI 48823, United States; CSIRO Land and Water, Ecosciences Precinct, 41 Boggo Road, QLD 4102, Australia; Venthic Technologies, Kipoupoleos 129, Peristeri, Athens 12137, Greece; PATH 2201 Westlake Avenue, Suite 200, Seattle, WA 98121, United States; Schools of Public Health and Earth and Biological Sciences, Loma Linda University, Loma Linda, CA 92350, United States; Department of Fisheries and Wildlife, Michigan State University, East Lansing, MI 48824, United States

**Keywords:** Geographic Information Systems (GIS), wastewater-based epidemiology, SARS-CoV-2, open data, public health, COVIDPoops19 dashboard

## Abstract

A year since the declaration of the global coronavirus disease 2019 (COVID-19) pandemic, there were over 110 million cases and 2.5 million deaths. Learning from methods to track community spread of other viruses such as poliovirus, environmental virologists and those in the wastewater-based epidemiology (WBE) field quickly adapted their existing methods to detect SARS-CoV-2 RNA in wastewater. Unlike COVID-19 case and mortality data, there was not a global dashboard to track wastewater monitoring of SARS-CoV-2 RNA worldwide. This study provides a 1-year review of the “COVIDPoops19” global dashboard of universities, sites, and countries monitoring SARS-CoV-2 RNA in wastewater. Methods to assemble the dashboard combined standard literature review, Google Form submissions, and daily, social media keyword searches. Over 200 universities, 1400 sites, and 55 countries with 59 dashboards monitored wastewater for SARS-CoV-2 RNA. However, monitoring was primarily in high-income countries (65%) with less access to this valuable tool in low- and middle-income countries (35%). Data were not widely shared publicly or accessible to researchers to further inform public health actions, perform meta-analysis, better coordinate, and determine equitable distribution of monitoring sites. For WBE to be used to its full potential during COVID-19 and beyond, show us the data.

## Teaser

Analysis of a global dashboard of COVID-19 wastewater monitoring indicates that most monitoring is in High Income Countries and there is a need for great equity and open data sharing.

## Introduction

In 1 year, the coronavirus disease 2019 (COVID-19) pandemic has resulted in 110 million cases and 2.5 million deaths worldwide (Dong et al. 2020). When the novel coronavirus strain (SARS-CoV-2) that causes COVID-19 emerged in late 2019, environmental virologists began rapidly adapting their methods from those supporting surveys of other pathogens within wastewater (GWPP 2021), including the use of public health elements to address concerns associated with monitoring SARS-CoV-2 RNA in wastewater. Some of the first major monitoring efforts for SARS-CoV-2 in wastewater were in the Netherlands (Medema et al. 2020a, Lodder and de Roda Husman 2020), Australia (Ahmed et al. 2020), Italy (La Rosa et al. 2020), and the USA (Sherchan et al. 2020). A global coordination effort was proposed to share and standardize sampling strategies, virus recovery methodologies, and data for wastewater-based epidemiology (WBE) for SARS-CoV-2 (Bivins et al. 2020). COVID-19 WBE and environmental/wastewater surveillance or monitoring are being used to describe this effort, which has grown from just a few countries in March 2020 to at least 55 countries and over 200 universities a year later (Naughton et al. 2021).

Both the growth and recognition of WBE for SARS-CoV-2 monitoring has been rapid and widespread. Wastewater monitoring to address epidemiological questions has been used historically at mostly smaller scale settings to track enteric viruses and other pathogens (GWPP 2021), including the poliovirus vaccine and wildtype strains (Hovi et al. 2011), norovirus, adenovirus, and other pathogens (Ali et al. 2021), as well as antimicrobial resistance (Hendriksen et al. 2019), and drugs such as opioids (Burgard et al. 2014, Li et al. 2019, Schmidt 2020). Because of the COVID-19 pandemic, a year later, at least seven countries, Finland (THL 2021), France (Obépine 2021), Hungary (NNK 2021), Luxembourg (LIST 2021), Netherlands (Rijksoverheid 2021), Spain (VATar 2021), and Turkey (Kocamemi et al. 2020), had nationalized wastewater monitoring for SARS-CoV-2. The USA (CDC 2021) and Canada (CWN 2021) established national coordination networks/systems. At least four countries have regional level monitoring: Australia (Queensland 2021, Victoria 2021), Brazil (ANA 2021), South Africa (SAMRC 2021), Switzerland (EAWAG 2021a;, EAWAG 2021b), and the UK (SEPA 2021). Throughout these countries and globally, newspaper, online, and television outlets have extensively covered SARS-CoV-2 wastewater monitoring with local to national level politicians calling for widespread application of wastewater testing. Whereas COVID-19 case and death data have been widely available globally, such as through the Johns Hopkins University dashboard (Dong et al. 2020), even the locations of COVID-19 wastewater testing are less available and difficult to track.

Though challenges exist to standardize wastewater testing methods and data normalization (Medema et al. 2020b), public health departments (CWN 2020), utilities, scientists, and engineers have an ethical obligation, especially during a pandemic, to provide this information to the public who is being monitored. The goal of this study is to provide a global dashboard and a 1-year analysis of SARS-CoV-2 wastewater testing to inform the public (general population, public health departments, municipalities, and researchers) where this type of testing is taking place and to provide links to available data for decision making and better coordination. Our hypothesis was that much of the wastewater SARS-CoV-2 data will not be publicly available and low- and middle-income countries would have less access to wastewater monitoring. This study uses the “COVIDPoops19” dashboard to identify gaps in wastewater monitoring to make recommendations for science communication of wastewater data, and as a call to action for more forthcoming and transparent open data sharing.

## Materials and methods

### Data sources

To create a global dashboard of reported wastewater monitoring efforts, six different data sources were used: (1) the COVID-19 WBE website (COVID-19 WBE Collaborative 2021), (2) webinars, (3) Google Form submissions, (4) literature searches, (5) Twitter keyword searches, and (6) Google keyword searches. ArcGIS Online Dashboards was chosen as the host platform (ESRI 2020). First, points were added from the COVID-19 WBE collaborative publication map as country points (COVID-19 WBE Collaborative 2021). A link to a Google Form was made available at the bottom of the COVIDPoops19 dashboard for users to submit public data points. A Twitter account (@COVIDPoops19) was created for the dashboard and the UC Merced co-authors performed key word searches daily for six combinations of “wastewater” or “sewage” and “COVID19” or “COVID-19” or “SARS-CoV-2.”

From advertisements on Twitter and the US National Science Foundation (NSF) COVID-19 WBE Research Coordination Network (RCN) (Wastewater Surveillance RCN 2021), the co-authors regularly attended webinars to learn about different monitoring efforts. Only publicly reported locations and data from websites and news articles were added to the dashboard. Google was used to check for missing US states and territories. For example, a combination of “Puerto Rico” and “wastewater,” “sewage,” “monitoring” and “COVID-19” and “SARS-CoV-2” keywords were used to see whether there were missing articles that were not found by the daily keyword searches on Twitter.

Although keyword and literature searches were predominantly in English, the dashboard team included English, French, and Spanish speakers, and the dashboard had a broad submission from international stakeholders via the Google form as well as engagement during international webinars. Many researchers in other countries also publish and post in English. Through the co-authors’ involvement in the W-SPHERE (Wastewater SARS Public Health Environmental REsponse) global data center (W-SPHERE 2021), working group, and Technical Advisory Committee; we were also able to directly engage with those monitoring in low- and middle-income countries.

### Dashboard curation

**Figure 1.**
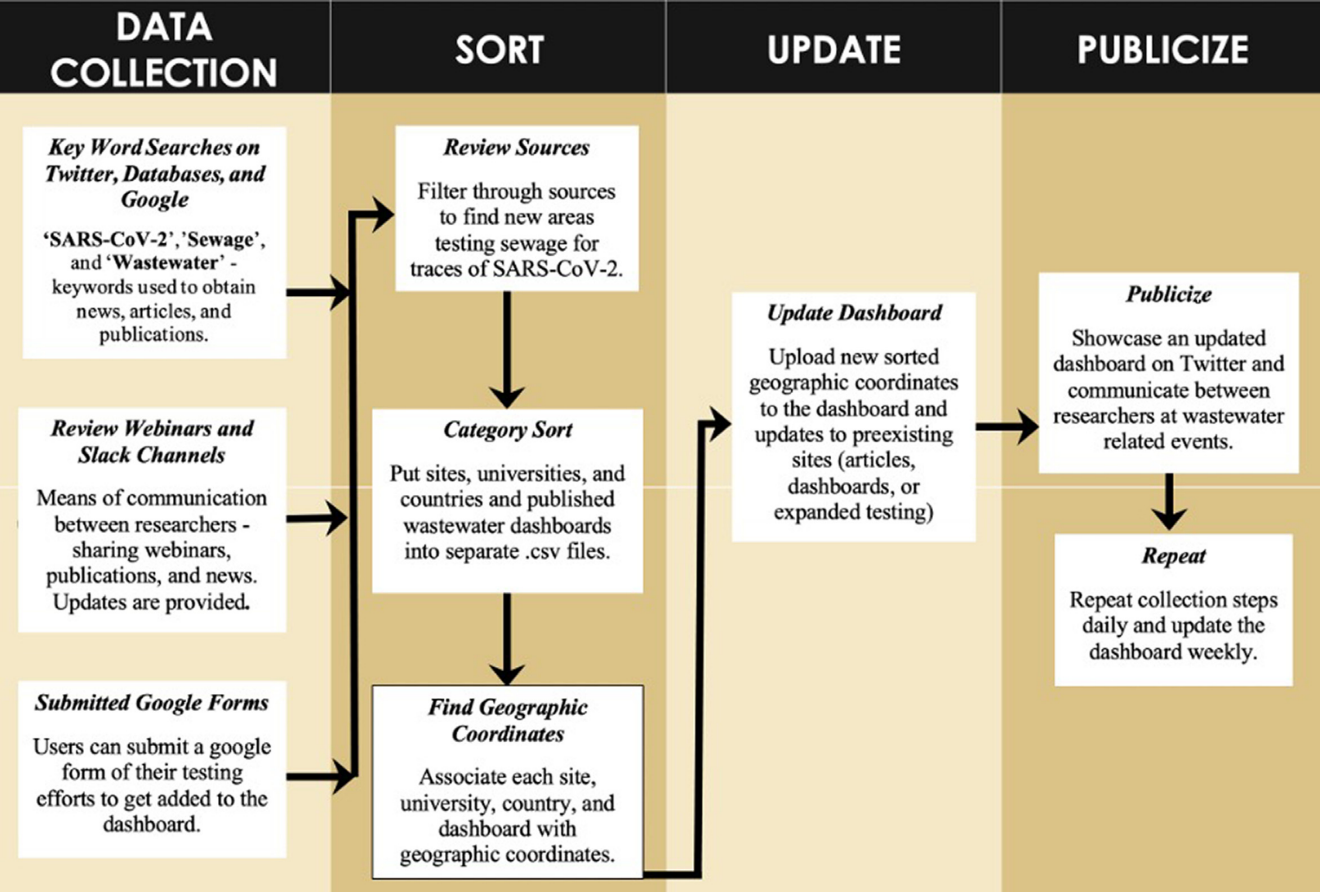
COVIDPoops19 dashboard data workflow.

Wastewater monitoring locations for SARS-CoV-2, news articles, publications, Google Form submissions, dashboard/data, and other web links were collected and sorted into the following four categories: (1) dashboard/data, (2) university, (3) country, and (4) sites (see Fig. 1). GPS coordinates in WGS 84 coordinate system for the dashboard were either directly extracted when provided or approximated from the location mentioned in the source. If a city, county, or country was found testing their wastewater for SARS-CoV-2 without specific sampling sites mentioned, then a point was placed near the centroid of the mentioned area tested to associate the testing site with a location. When other public dashboards for wastewater testing efforts provided coordinates for their sampled sites, those were downloaded and utilized as site points on the COVIDPoops19 dashboard. The COVIDPoops19 dashboard was usually updated weekly depending on the number of points gathered and submitted.

### Data analysis

After the collection of sites, universities, and countries, the spatial distribution of wastewater monitoring was analyzed. Countries were sorted based on the World Bank income classifications (high-income countries (HICs), upper middle-income countries, lower middle-income countries (LMIC), and low-income countries) (World Bank 2021). ArcGIS Pro 2.6.1 was used to map the number of sites and universities monitoring wastewater for SARS-CoV-2 globally. With a large number of sites and universities monitoring SARS-CoV-2 in wastewater, the USA was chosen to further classify based on the 50 states and 5 inhabited territories.

Dashboards were categorized based on their presentation, communication style, and data availability. Results of SARS-CoV-2 testing in wastewater were presented as maps, graphs, a small written description, or solely by color (demonstrating an increase or decrease in trend). Dashboard communication style categories were video, FAQ page, a short written format (less than three paragraphs), longer descriptions (three or more paragraphs), and no form of written communication. The simplicity of the communication was also determined by whether the description given was: (1) technical, more specifics on the science behind SARS-CoV-2 wastewater testing (included information on lab processes), or (2) a simpler form of communication that would be understandable to the general public (used general vocabulary to inform as to why wastewater is being employed to test for SARS-CoV-2). Dashboards were checked for whether they provided downloadable data, the file type, and the variables available. Data on population monitored, flow rate, gene targets, methods, study duration, sampling frequency, and other variables are not available for many dashboards and sites and this information could not be included in our analysis.

## Results and discussion

As of 11th March 2021, a year after declaration of the COVID-19 pandemic (Cucinotta and Vanelli 2020), the COVIDPoops19 global dashboard for wastewater monitoring of SARS-CoV-2 included 235 universities, 59 dashboards, and 1488 sites in 55 countries. Between September 2020 and 11th March 2021, there were 60 submissions on the Google Form linked to the COVIDPoops19 dashboard. Since the dashboard was published publicly in September 2021, there have been 25 679 visits. The COVIDPoops19 twitter account has acquired over 2000 followers between May 2020 and March 2021.

### Wastewater monitoring equity analysis

**Figure 2.**
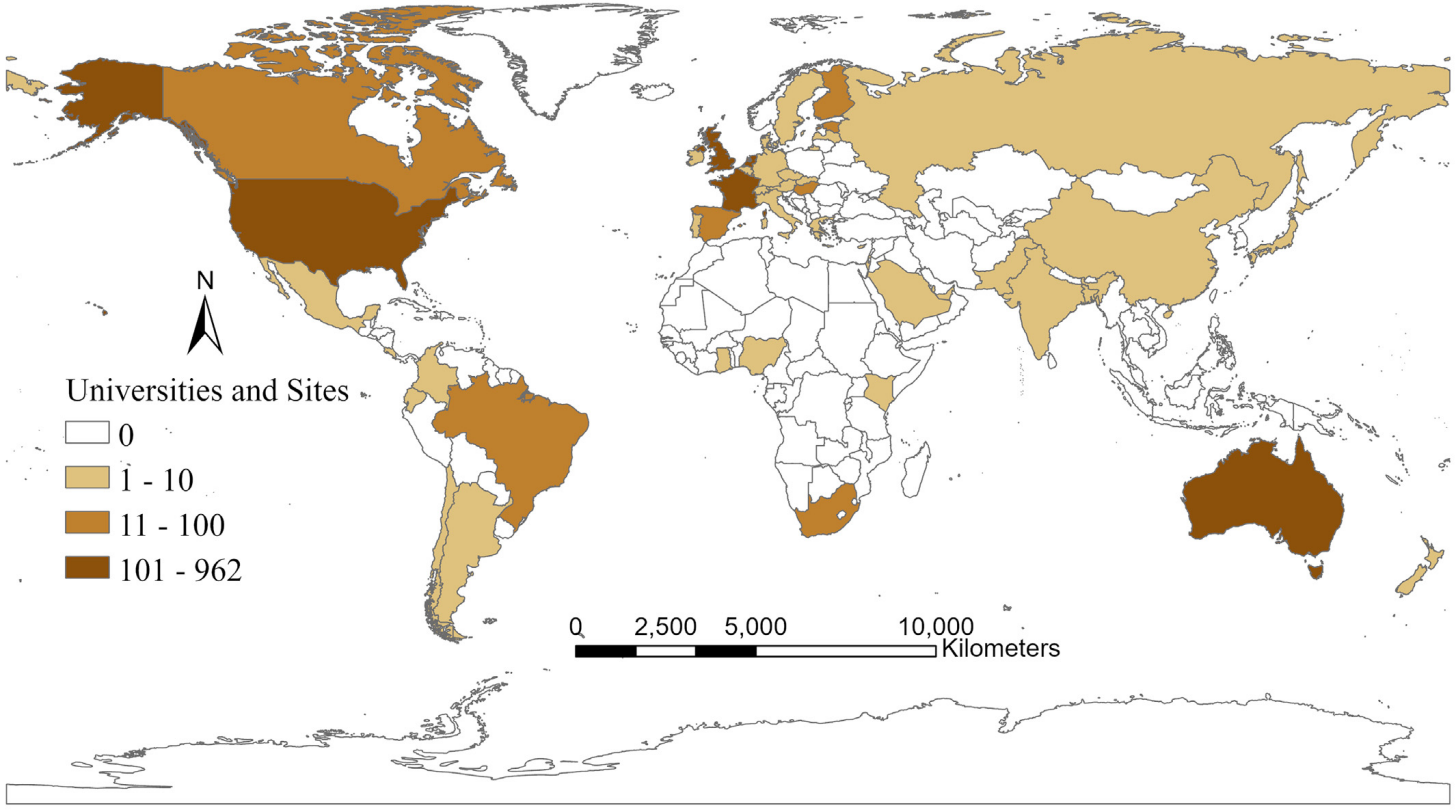
World map with countries using wastewater monitoring of SARS-CoV-2.

Of the 195 countries in the world (US DOS 2021), 55 contain wastewater monitoring. Of these 55, 36 (65%) are in HICs, 11 (20%) are upper middle-income countries, 8 (15%) are LMICs, and 0% are low-income countries (Fig. 2). Similar to COVID-19 individual testing and Personal Protective Equipment (PPE; Kavanagh et al. 2020, McMahon et al. 2020) and vaccination efforts (Lancet Commission 2021), access to wastewater testing is also more widely available in HICs.

**Figure 3.**
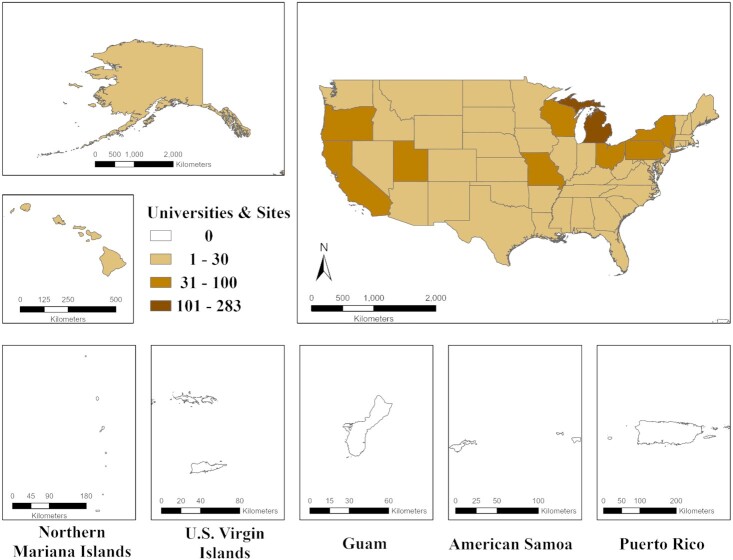
Map of the USA and US-inhabited territories testing wastewater for SARS-CoV-2 RNA.

The USA had the highest number of universities and sites (962) monitoring for SARS-CoV-2 globally. Of the 50 states and 5 inhabited territories of the USA and the District of Columbia, there was no record of wastewater testing for SARS-CoV-2 RNA in any of the inhabited territories within the USA. The five inhabited territories were: (1) American Samoa, (2) Guam, (3) Puerto Rico, (4) US Virgin Islands, and (5) Northern Mariana Islands (see Fig.   3). Iowa had no publicly disclosed wastewater testing until the University of Iowa added testing in February 2021 (University of Iowa 2021). South Dakota had only one location monitoring for SARS-CoV-2 RNA since July 2020. Greater distribution of wastewater monitoring for SARS-CoV-2 would be beneficial in the USA and US-inhabited territories since WBE has potential as an early warning system and to identify hotspots to better target public health measures to prevent further COVID-19 cases (Ahmed et al. 2021).

### Show us the data

The COVIDPoops19 dashboard is the most extensive compilation of global wastewater monitoring for SARS-CoV-2. The dashboard is likely an underestimate of the locations testing wastewater for SARS-CoV-2 RNA, because it is limited to publicly available data. Many private companies who are monitoring wastewater for SARS-CoV-2 are limited by what their client(s) (e.g. public health department, municipality, etc.) allow to be shared. For example, Biobot Analytics is a private company that conducts WBE (Biobot Analytics 2021a). Biobot has processed wastewater from at least 300 sites in 42 states in the USA (Wiggins 2020). Some Biobot sites were found and posted from news articles and publicly available dashboards, e.g. Eastern Massachusetts (Biobot Analytics 2021b), Chattanooga (Biobot Analytics 2021c), Nantucket (Town and Country of Nantucket 2021), Delaware (Biobot Analytics 2021d), etc., but the COVIDPoops19 dashboard was missing other sites. In May 2021, Biobot did release aggregate data (Biobot Analytics 2021e), over a year into monitoring. Similarly, universities do not publicly report all the sites they are sampling from.

Despite over 200 universities, 1400 sites, and 55 countries with reported wastewater surveillance for SARS-CoV-2, there are a limited number of entities that make their data openly accessible with only 59 publicly available dashboards. Of these 59 dashboards, only 18 had downloadable data for further analysis (see Supplementary Information Table S1). Data are downloadable as .csv, .xlsx, .rda, .pdf, or .pitemx files depending on the dashboard. Typical data include flow rates, collection dates, coordinates, days since sampled, sample types, gene copy information, and if the virus was observed in the sample. Data available and units vary for each dashboard, as there is no common data standard followed among the different endeavors.

It is essential to ensure appropriate public health surveillance systems and open data access in pandemic response. The Canadian Water Network states, “During a public health emergency, it is imperative that all parties involved in surveillance share data in a timely fashion.” (CWN 2020). Providing open access to data collected from testing wastewater for SARS-CoV-2 RNA, along with effective communication and properly handling sensitive information, can better inform the public, which will allow for a collective fight against the COVID-19 pandemic.

Increased access to wastewater testing data could provide other researchers, such as data scientists, the opportunity to further develop algorithms, compare between sites, and better analyze the data to make it more useful to inform public health decisions instead of keeping it internal. Individuals may use wastewater data in their personal risk decisions if they see an increase in concentrations in the wastewater in their area or where they may travel. We have seen the general public regularly using and tweeting about the wastewater data on Twitter and from news media reports. However, an ongoing challenge of WBE is the lack of normalization across datasets. This is a nascent research space with high variability in methods used to collect, process, and analyze samples.

Increased data sharing may allow for analysis across collection sites and identifying which methods work best in HIC and LMIC settings (Pandey et al. 2021). Wastewater testing could be a useful and cost effective option in low-resource settings with limited clinical testing (Hart and Halden 2020, Usman et al. 2020). Greater open data would also facilitate better collaboration, coordination, and equity analysis. Most testing is concentrated in HICs. However, even within HICs, there may be inequity in distribution to high-income and urban areas with less diversity similar to disparities in individual testing (Hopper et al. 2020) and vaccination (Ndugga et al. 2021) in the USA.

The US National Wastewater Surveillance System (NWSS) currently only allows access to the wastewater concentration data on their internal dashboard to public health departments (CDC 2021). Nearly 2 years into the pandemic, the US CDC released a dashboard of their wastewater SARS-CoV-2 data on their COVID Data Tracker (CDC 2022). The wastewater data on CDC’s COVID Data Tracker show the 15-day percent change or 15-day detection proportion. Wastewater concentration data must be requested through the CDC. Not all wastewater monitoring in the USA is reported to CDC. Previously, the US Health and Human Services (HHS) aimed to test 30% of the US population through wastewater (Genomeweb 2020). HHS has yet to publicly release the locations where wastewater sampling occurred. Without knowing all the locations, researchers, the media, and the general public have no way to determine whether wastewater testing is equitably distributed among the 50 states, territories, and low-income, minority, and rural communities.

The USA has an OPEN (Open, Public, Electronic, and Necessary) Government Data Act that mandates federal agencies to make their data open (Data.gov 2021). A total of 53 other countries that also have open data websites and policies are listed on Data.gov. The European Council prioritized the adoption of Open Science and reusability of research data, promoting FAIR (Findable, Accessible, Interoperable, and Reusable) data principles (Mons et al. 2017). COVID-19 case and death data have been invaluable during the pandemic to inform the public and policies. Wastewater data can be aggregated and de-identified similar to case, hospitalization, and death data to protect private health information.

### Dashboard communication styles

A total of 59 dashboards were categorized on how their results were primarily presented. A total of 28 (47%) presented their dashboards in the form of a map, 28 (47%) used graphs, 2 (3%) solely gave a written description of the results (Erie County 2021, Lewis and Clark County 2021), and 1 (2%) presented an image with a color to demonstrate the trend (Indiana Borough 2021). A total of 14 (24%) dashboards used both a graph and a map, and 18 dashboards (30%) used colors to visually present results.

Of the 59 dashboards, 14 (34%) had no description of the data provided. Of the 45 dashboards that had some form of description, 25 (56%) dashboards used a short written format, 13 (29%) included more than three written paragraphs, 9 (20%) included a Frequently Asked Questions (FAQ) page, and 3 (7%) had videos. Five dashboards (13%) used a combination of communication styles. Valencia, Spain had a video and included multiple paragraphs (GoAigua 2020). New Haven, Connecticut, and Bozeman, Montana, both had a video and a short written format to describe SARS-CoV-2 testing in wastewater (Yale University 2020, Healthy Gallatin 2021), and the Luxembourg and Missouri dashboards have a short written format and a section with FAQ (LIST 2021, Missouri Department of Health and Senior Services 2021). Lastly, of the dashboards that presented some form of communication, 34 (76%) were written in language that could be understood by the general public, whereas 11 (24%) had very specific and detailed scientific information. For example, the dashboard used for Valencia, Spain had a simple communication style for a general audience to understand how wastewater can be used as a tool to better understand COVID-19 trends in their area (GoAigua 2020). In contrast, the dashboard used for Minas Gerais, Brazil, went more in-depth with the scientific specifics of the lab results and was categorized as more technical (ANA 2021).

While offering detailed and technical information about the wastewater testing process/protocol is ideal, it is also important to communicate the benefits of wastewater testing for the general public. For this reason, successful communication styles should include more understandable vocabulary (e.g. less scientific jargon) with links to WBE case studies, while offering links to more detailed information for more technical audiences (e.g. researchers, public health departments, and municipalities). Additionally, providing a video explanation can help more visual learners.

### Dashboard recommendations

Table 1 includes a list of recommendations for and benefits of public sharing of wastewater monitoring data based on our experience through the COVIDPoops19 dashboard, W-SPHERE global data center (W-SPHERE 2021), and participation in the National Science Foundation Funded COVID-19 Wastewater Based Epidemiology Collaborative (COVID-19 WBE Collaborative 2021) and Research Coordination Network (Wastewater Surveillance RCN 2021).

**Table 1.** Recommendations for publicly sharing COVID-19 wastewater monitoring data.

Considerations	Recommendations	Benefits
Data accessibility	Report data collected in a highly accessible manner that is open to the public. Follow the FAIR (Findable, Accessible, Interoperable, and Reusable) principles (Mons et al. 2017)	Provides effective communication between public health officials and citizens, helping inform decision making
Methodology	Explain methods of sampling processes and lab analysis	Allows for easy interpretation of the data considering the sampling process and extraction methodology varies between laboratories
Results	Detail how to interpret results and their significance	Enables the viewer to make informed decisions while understanding the limitations of wastewater monitoring
Downloadable data	Provide viewers with easily downloadable data and distinguishable units on a project website and/or open data repository or center (e.g. GitHub, NORMAN Score Database (Norman Score Database, 2021), Canadian Open Data Model (PHES-ODM, 2021), and/or W-SPHERE)	Allows for public sharing and open access to data for innovation in research to identify trends across the community and to prepare for future outbreaks. Open data repositories and centers often have unique identifiers to cite the data in further publications
Communications	Define goal and scope of the project tailored for a wide audience. Include short videos for more visual learners and avoid scientific jargon	Educates the public through transparency and open communication
Surveillance level and location	Present some degree of spatial coverage affected by the conducted wastewater testing	Distinguishes hierarchy of community-level surveillance and methodology required to analyze samples
Parameters	Report all parameters within the sampling location that may impact results: time, targeted genes, flow, dates (sampling and processing), population served, etc. (McClary-Gutierrez et al. 2021)	Identifies parameters within the sampling location for most accurate data interpretation

For WBE to be used to its full potential as a public health tool during and after the COVID-19 pandemic, data must be more openly shared with the public and among researchers. Wastewater monitoring and support for dashboard development must also be expanded to lower-income countries and areas. Wastewater monitoring will remain important throughout vaccination efforts to monitor for outbreaks (Smith et al. 2020) and can be used to track the spread of variants at larger scales (Martin et al. 2021), only if they show us the data.

## Supplementary Material

xtad003_Supplemental_Files

## Supplementary information

Table of wastewater dashboards for COVID-19 and whether they included downloadable data.
