# Supplementary material for: Show us the data: global COVID-19 wastewater monitoring efforts, equity, and gaps
Source: FEMS Microbes. 2023 Jan 12;4:xtad003. doi: 10.1093/femsmc/xtad003 (PMC10117741; doi:10.1093/femsmc/xtad003)
Supplement: xtad003_Supplemental_Files [file xtad003_supplemental_files.zip › SupplInfo_Showusdata_12_13_22_clean.docx]

**Supporting Information**

**Show us the Data: Global COVID-19 Wastewater Monitoring Efforts, Equity, and Gaps**

Colleen C. Naughton*^1^, Fernando A. Roman, Jr.^1^, Ana Grace F. Alvarado^1^, Arianna Q. Tariqi^1^, Matthew A. Deeming^1^, Krystin F. Kadonsky^1^, Kyle Bibby^2^, Aaron Bivins^2^, Gertjan Medema^3,4,5^, Warish Ahmed^6^, Panagis Katsivelis^7^, Vajra Allan^8^, Ryan Sinclair^9^, Joan B. Rose^10^

*Corresponding Author [cnaughton2@ucmerced.edu](mailto:cnaughton2@ucmerced.edu)

^1^Department of Civil and Environmental Engineering, University of California at Merced, Merced, CA 95343, USA

^2^Department of Civil & Environmental Engineering & Earth Science, University of Notre Dame, 156 Fitzpatrick Hall, Notre Dame, IN, 46556, USA.

^3^KWR Water Research Institute, Groningenhaven 7, Nieuwegein, 3433 PE, the Netherlands

^4^Delft University of Technology, Stevinweg 1, Delft, 2628 CN, the Netherlands

^5^Michigan State University, 1405 S Harrison Rd, East-Lansing, Michigan, 48823, USA

^6^CSIRO Land and Water, Ecosciences Precinct, 41 Boggo Road, QLD 4102, Australia.

^7^Venthic Technologies, Kipoupoleos 129, Peristeri, Athens, Greece

^8^PATH 2201Westlake Avenue, Suite 200 Seattle, WA 98121, USA

^9^Schools of Public Health and Earth and Biological Sciences, Loma Linda University Loma Linda, CA 92350, USA

^10^Department of Fisheries and Wildlife, Michigan State University, East Lansing, Michigan 48824, USA

**Table of Contents**

| **Table S1:** List of dashboards/data for wastewater monitoring of SARS-CoV-2 | …………..S2 |
| --- | --- |

**Supporting Information Table S1:** List of dashboards/data for wastewater monitoring of SARS-CoV-2

| N | Country | City | State | Latitude | Longitude | Website | Downloadable Data? |
| --- | --- | --- | --- | --- | --- | --- | --- |
| 1 | United States | Athens | Georgia | 33.9519 | -83.3576 | https://lipplab-uga.github.io/covid_wastewater_lipplab_athens/ | Yes |
| 2 | United States | New Haven | Connecticut | 41.3083 | -72.9279 | https://yalecovidwastewater.com/ | No |
| 3 | United States | Clemson | South Carolina | 34.6834 | -82.8374 | https://www.clemson.edu/covid-19/testing/wastewater-dashboard.html | No |
| 4 | United States |  | Utah | 39.321 | -111.09 | https://deq.utah.gov/water-quality/sars-cov-2-sewage-monitoring | No |
| 5 | United States | Tempe | Arizona | 33.4255 | -111.94 | https://covid19.tempe.gov/ | Yes |
| 6 | United States |  | Massachusetts | 42.3506 | -70.9577 | http://www.mwra.com/biobot/biobotdata.htm | Yes |
| 7 | United States |  | Ohio | 40.4173 | -82.9071 | https://coronavirus.ohio.gov/wps/portal/gov/covid-19/dashboards/wastewater | Yes |
| 8 | United States | New Castle | Delaware | 39.6955 | -75.6734 | http://biobot.newcastlede.gov/ | No |
| 9 | United States | Boise | Idaho | 43.615 | -116.2023 | https://www.cityofboise.org/departments/mayor/coronavirus-covid-19-information/covid-19-data/wastewater-testing/ | Yes |
| 10 | United States | Bozeman | Montana | 45.677 | -111.0429 | https://www.healthygallatin.org/coronavirus-covid-19/wastewater-data/ | No |
| 11 | Netherlands |  |  | 52.1326 | 5.2913 | https://coronadashboard.rijksoverheid.nl/ | Yes |
| 12 | Spain | Valencia |  | 39.4699 | -0.3763 | https://www.idrica.com/blog/valencia-anticipates-coronavirus-outbreaks-thanks-to-goaigua-sars-analytics/ | No |
| 13 | Canada | Ottawa | Ontario | 45.4215 | -75.6972 | https://613covid.ca/wastewater/# | Yes |
| 14 | Hungary |  |  | 47.1683 | 19.5032 | https://www.nnk.gov.hu/index.php/koronavirus-tajekoztato/743-jelentos-emelkedes-a-megbetegedesek-szamaban-a-kovetkezo-1-2-hetben-tovabbra-sem-varhato | No |
| 15 | Finland |  |  | 61.9241 | 25.7482 | https://www.thl.fi/episeuranta/jatevesi/jatevesiseuranta_viikkoraportti.html | Yes |
| N | Country | City | State | Latitude | Longitude | Website | Downloadable Data? |
| 16 | United States | Gilbert | Arizona | 33.3528 | -111.789 | https://experience.arcgis.com/experience/19a447509ff44fcf922ad0892b7b2cce/page/page_21/ | No |
| 17 | United Kingdom |  | Scotland | 56.8169 | -4.1827 | https://informatics.sepa.org.uk/RNAmonitoring/ | Yes |
| 18 | Switzerland | Zurich |  | 47.3961 | 8.545548 | http://parsivel-eawag.ch/sarscov2/ARA_Werdhoelzli_ddPCR.html | Yes |
| 19 | United States | Corvallis | Oregon | 44.5646 | -123.262 | http://people.oregonstate.edu/~levit/Software/CorvallisWastewater102120.html | No |
| 20 | Australia |  | Victoria | -37.809 | 144.970 | https://www.dhhs.vic.gov.au/wastewater-monitoring-covid-19 | No |
| 21 | Brazil |  | Rio Grande do Sul | -29.186 | -51.170 | https://app.powerbi.com/view?r=eyJrIjoiMzNhZjNiNjUtZjQxZS00ZDA0LWFhZTctNjNlNjg0Mjg5Y2FiIiwidCI6IjE1ZGNkOTA5LThkYzAtNDBlOS1hMWU1LWNlY2IwNTNjZGQxYSJ9&pageName=ReportSection | No |
| 22 | South Africa |  |  | -33.924 | 18.4232 | <https://www.samrc.ac.za/wbe/> | No |
| 23 | Luxembourg |  |  | 49.6101 | 6.130619 | <https://www.list.lu/en/covid-19/coronastep/> | No |
| 24 | United States | Indiana | Pennsylvania | 40.6231 | -79.155 | <https://www.indianaboro.com/news/categories/wastewater-surveillance> | No |
| 25 | Spain |  | Catalonia (region) | 41.983 | 2.82493 | <https://sarsaigua.icra.cat/> | Yes |
| 26 | United States | Nantucket (Island) | Massachusetts | 41.2667 | -70.0158 | <https://nantucket-ma.gov/1864/Surfside-Wastewater-Treatment-Facility-C> | No |
| 27 | United States | Keene | New Hampshire | 42.9263 | -72.2832 | <https://www.keene.edu/featured/fall2020/covid-19-dashboard/> | No |
| 28 | United States | Burlington | Vermont | 44.4880 | -73.2080 | <https://coronavirus-response-burlingtonvt.hub.arcgis.com/pages/wastewater-monitoring> | No |
| N | Country | City | State | Latitude | Longitude | Website | Downloadable Data? |
| 29 | United States |  | Oregon | 44.0578 | -120.7331 | <https://public.tableau.com/profile/oregon.health.authority.covid.19#!/vizhome/OregonsSARS-CoV-2WastewaterMonitoring/WastewaterDashboard> | No |
| 30 | United States | Jupiter | Florida | 26.9245 | -80.10546 | <https://loxahatcheeriver.org/wastewater-surveillance/> | No |
| 31 | Australia |  | Queensland | -21.631 | 148.03938 | <https://www.qld.gov.au/health/conditions/health-alerts/coronavirus-covid-19/current-status/wastewater> | No |
| 32 | United States |  | Wisconsin | 43.7844 | -88.7879 | https://www.dhs.wisconsin.gov/covid-19/wastewater.htm | Yes |
| 33 | Brazil |  | Minas Gerais | -19.912 | -44.0282 | <https://t.co/RY4D877qeW?amp=1> | No |
| 34 | United States | Erie | Pennsylvania | 42.1292 | -80.0851 | <https://eriecountypa.gov/tag/biobot/> | No |
| 35 | United States |  | Michigan | 44.7648 | -84.7052 | <https://egle.maps.arcgis.com/apps/webappviewer/index.html?id=8e3cb66aca204876a1db0e1b663af805> | Yes |
| 36 | United States | Chattanooga | Tennessee | 35.0528 | -85.3330 | <https://connect.chattanooga.gov/covid-biobot-analysis-reports/> | No |
| 37 | United States |  | Pennsylvania | 41.2892 | -77.44583 | <https://www.cor.pa.gov/Pages/COVID-19.aspx> | No |
| 38 | United States | Southeastern | Virginia | 37.0299 | -76.3452 | https://www.hrsd.com/HRSD-COVID-19-Surveillance | No |
| 39 | United States |  | Colorado | 39.7392 | -104.9903 | https://cdphe.maps.arcgis.com/apps/opsdashboard/index.html#/d79cf93c3938470ca4bcc4823328946b | Yes |
| 40 | United States |  | Illinois | 41.6441 | -88.450 | [https://portal.rjngroup.com/arcgisportal/apps/opsdashboard/index.html#/594d4b1b2dd840958cedb50b1381982b](https://portal.rjngroup.com/arcgisportal/apps/opsdashboard/index.html#/594d4b1b2dd840958cedb50b1381982b ) | No |
| 41 | United States |  | Missouri | 38.5762 | -92.175 | <https://storymaps.arcgis.com/stories/f7f5492486114da6b5d6fdc07f81aacf> | No |
| 42 | Switzerland | Lausanne | Vaud | 46.5244 | 6.59186 | <https://sensors-eawag.ch/sarscov2/STEP_Vidy_ddPCR.html> | Yes |
| 43 | United States | Cambridge | Massachusetts | 42.3742 | -71.1165 | <https://cityofcambridge.shinyapps.io/COVID19/#shiny-tab-wastewater> | No |
| N | Country | City | State | Latitude | Longitude | Website | Downloadable Data? |
| 44 | United States |  | New Mexico | 32.631 | -105.86 | <https://www.env.nm.gov/wastewater-surveillance-system-data-dashboard/> | Yes |
| 45 | Canada | Windsor | Ontario | 42.296 | -83.0255 | <https://wechu.org/cv/weekly-epidemiological-summary> | No |
| 46 | United States | San Diego | California | 32.8797 | -117.229 | <https://returntolearn.ucsd.edu/dashboard/index.html> | No |
| 47 | United States | Helena | Montana | 46.590 | -112.019 | <https://www.lccountymt.gov/health/covid-19/local-covid-19-decision-making-dashboard.html> | No |
| 48 | USA | Mansfield | Connecticut | 41.8114 | -72.264 | <https://covid-testing.uconn.edu/dashboard/> | No |
| 49 | France |  |  | 46.601375 | 2.3095 | <https://www.reseau-obepine.fr/donnees-ouvertes/> | No |
| 50 | Canada | Calgary | Alberta | 50.9940 | -114.071 | <https://www.chi-csm.ca/> | No |
| 51 | Spain | Madrid |  | 40.4200 | -3.7019 | <https://www.canaldeisabelsegunda.es/mapa-vigia> | No |
| 52 | Canada |  | Northwest Territories | 64.8255 | -124.84 | <https://nwt-covid.shinyapps.io/Testing-and-Cases/?lang=1> | No |
| 53 | Sweden | Uppsala |  | 59.8586 | 17.69389 | <https://crush-covid.shinyapps.io/crush_covid/> | No |
| 54 | United States | Telluride | Colorado | 37.9375 | -107.81 | https://sanmiguelco.maps.arcgis.com/apps/opsdashboard/index.html#/56e682135d1d4128bee1a0426aed1d10 | Yes |
| 55 | Canada |  | Metro Vancouver | 49.2501 | -123.08 | http://www.metrovancouver.org/services/liquid-waste/environmental-management/covid-19-wastewater/Pages/default.aspx | No |
| 56 | United States | Honolulu | Hawaii | 21.3069 | -157.85 | https://www.oneoahu.org/dashboard | Yes |
| 57 | Spain |  |  | 40.4637 | -3.7492 | https://miteco.maps.arcgis.com/apps/opsdashboard/index.html#/aab0e0653d694289b310f6485f9f2226 | No |
| 58 | USA | St. Mary's County | Maryland | 38.3441 | -76.5764 | <http://smchd.org/covid-19-wastewater> | No |
| 59 | Spain | Edar Bens | A Coruna | 43.3677 | -8.4559 | Edarbens.es/covid19 | No |
