# Supplementary material for: Show us the data: global COVID-19 wastewater monitoring efforts, equity, and gaps
Source: FEMS Microbes. 2023 Jan 12;4:xtad003. doi: 10.1093/femsmc/xtad003 (PMC10117741; doi:10.1093/femsmc/xtad003)
Supplement: xtad003_Supplemental_Files [file xtad003_supplemental_files.zip › FEMSMC-2022-017 reviews and author response.docx]

**Reviews of manuscript FEMSMC-2022-017**

**Reviewer 1 - Ricardo Rosario dos Santos**

**Recommendation: Accept**

The manuscript "Show us the Data: Global COVID-19 Wastewater Monitoring Efforts, Equity,and Gaps" perfectly shows the importance of the systematic collection and interpretation of available data. The systematic compilation of data that was performed in this case for the presence of SARS-CoV-2 in wastewater should be mimic for other cases.

**Editor’s comments**

**Recommendation: Minor Revision**

We appreciate the authors patients with the review process. It has been challenging to find multiple reviewers therefore, to avoid further delays, I have also reviewed the manuscript and provide comments to the authors.

The manuscript nicely describes the reporting and monitoring of covid19 via the wastewater and it is a highly pertinent example for future monitoring and managing infectious diseases in society. I have a few minor comments.

- The methods section is a bit confusing, possibly due to that it is one section. It describes the source of data and data collection, whereas the data analysis appears to be less clear. If the data sources and collections are in a separate heading from the data analysis, this would improve clarity.

- Figure S1 describes the work flow and should be included in the main document in the methods, rather than as supplementary material. I believe it contributes valuable information on the data processing.

- The results and discussion could also be divided into several sub-sections for readability.

**Author's Response to Decision Letter for (FEMSMC-2022-017)**

Response to Reviewers

Associate Editor

“Comments to the Author:

We appreciate the authors patience with the review process. It has been challenging to find multiple reviewers therefore, to avoid further delays, I have also reviewed the manuscript and provide comments to the authors.

The manuscript nicely describes the reporting and monitoring of covid19 via the wastewater and it is a highly pertinent example for future monitoring and managing infectious diseases in society. I have a few minor comments.

- The methods section is a bit confusing, possibly due to that it is one section. It describes the source of data and data collection, whereas the data analysis appears to be less clear. If the data sources and collections are in a separate heading from the data analysis, this would improve clarity.

- Figure S1 describes the work flow and should be included in the main document in the methods, rather than as supplementary material. I believe it contributes valuable information on the data processing.

- The results and discussion could also be divided into several sub-sections for readability.”

Comment #1: “- The methods section is a bit confusing, possibly due to that it is one section. It describes the source of data and data collection, whereas the data analysis appears to be less clear. If the data sources and collections are in a separate heading from the data analysis, this would improve clarity.”

Response #1: We apologize that the methods were confusing. In our effort to be concise, some method detail may have been lost. We have added separate headings for “data sources”, “dashboard curation”, and “data analysis” as suggested by the reviewer. We moved up a paragraph on the “data curation” to flow better into the analysis. Additionally we added the work flow diagram from the supplementary information (see response #2) under the new “dashboard curation” sub-section in the methods.

Comment #2: “- Figure S1 describes the work flow and should be included in the main document in the methods, rather than as supplementary material. I believe it contributes valuable information on the data processing.”

Response #2: This is a great suggestion. Thank you. We have added the work flow Figure S1 into the main document in the methods under the “dashboard curation” subsection.

Comment #3: “The results and discussion could also be divided into several sub-sections for readability.”

Response #3: We have added several sub-sections, “Wastewater monitoring equity analysis”, “Show us the data”, “Dashboard communication Styles”, and “Dashboard recommendations” for readability. Sometimes journals do not permit many subsections. Thank you for the suggestion.

To further increase readability and flow with our new subsections as requested by the reviewer, we also moved up the U.S. analysis paragraph and Figure 3 to better fit with the “Wastewater monitoring equity analysis” section and shifted three paragraphs on dashboard communication analysis to the “Dashboard Communication Styles” section after the “Show us the Data section.”

Reviewer: 1

“Comments to the Author

The manuscript "Show us the Data: Global COVID-19 Wastewater Monitoring Efforts, Equity,and Gaps" perfectly shows the importance of the systematic collection and interpretation of available data. The systematic compilation of data that was performed in this case for the presence of SARS-CoV-2 in wastewater should be mimic for other cases.”

Response to Reviewer #1: We thank the reviewer for their kind words. We hope that our manuscript can be a model for other infectious diseases and cases (as remarked by another reviewer as well) and others can learn from our method. We are in the process of applying our method for other pathogens monitored in wastewater other than SARS-CoV-2 but that is outside the scope of this manuscript and still under development.
